# Case of Colloid Tumor in the Cavity of the Cranium, Presented to the Medical Department of the National Institute at Its Meeting on the First of July, 1844

**Published:** 1845-04

**Authors:** Thomas Miller


*Case of Colloid Tumor in the Cavity of the Cranium, Presented to the Medical Department of the National Institute at its Meeting on the the first of July*, 1844. By THOMAS MILLER, M.D.—G. C., Purser in the U. S. Navy, æt. 42, temperate habits, but has been much exposed from the nature of his occupation; many, say ten, years since, was effected with symptoms of dyspepsia, frequent violent pains in the back of his head, which rendered him irritable and unsettled in his temper, impatient, and often disposed to change his residence, &c. A few years after this, while at sea, he had violent and alarming attacks of cramp colic, and one or two violent and alarming hæmorrhages from his lungs. Six or seven years ago he was suddenly attacked with aphonia, for which, he was treated by numerous medical gentlemen of the Navy, and private practitioners both of this and other countries.

Twelve months ago I was called to attend him: he exhibited to me his left ear, from which protruded a foreign body, which had been pronounced to be a polypus. He stated that he had endured and was still enduring great suffering from his head, ear and throat, referred all his pain to the back part of his head, in each side over the junction of the occipital and temporal bones, extending posteriorly along the nucha and anteriorly along the course of the Eustachian tube and the large vessels of the neck—he could not, nor had he been able for many years, to lie easily on his left side.

The immediate cause of my being called was a severe hæmorrhage from his lungs; he had discharged a large quantity of light and frothy blood, and he continued to throw up the same, though in smaller quantities. With leeching, lead, &c., this hæmorrhage was readily controlled; in a few days, he had a similar attack, which was readily relieved by oil of turpentine in small doses. With some difficulty he went to Philadelphia, where he consulted Dr. Ruschenberger of the U. S. Navy. I was of the opinion that the blood, which he discharged, was from the lungs, and that he would ultimately have phthisis. In the month of August, 1843, Dr. Ruschenberger removed, by the forceps and caustic, the foreign body from the ear. He suffered much; it was supposed that every vestige of the body was removed. He remained under the charge of Dr. Ruschenberger and others, till the month of December, when he came to this city and placed himself under my charge. When sent for, I found him in the following condition, viz: he was lying in bed, complaining of great restlessness and general pain, particularly severe about the back of the head, neck, and in the mastoid portion of the left temporal bone; the pain extending along the course of the large vessels, and at the condyles and angles of the jaw (left side.) There was partial paralysis of his tongue, which was drawn to the left side, when an effort was made to protrude it. He had much difficulty in deglutition, often strangled while eating, and often coughing up what he had swallowed. There was a remarkable paleness of the left side of his tongue, while the right side, up to the median line, was more than usually red; pressure on the thyroid cartilage, &c. produced no pain. He complained constantly of an uneasiness in his chest and sense of stricture; not much cough; sense of distension and fulness in his abdomen, which was constricted. He entertained the idea that it was necessary to be purged frequently, and therefore took oil every day or two—a very small dose of which acted well on him. Nothing material was done for him, believing that his case was either irremediable, or was to be left to time. "He was under the impression that there was a foreign substance in his ear, and in the cells of the mastoid process; and that his pneumogastric nerve was affected, and that this foreign substance had passed to the throat."

He continued in this condition, losing flesh and strength, constantly predicting that he must soon die, and that nothing would benefit him. He had frequent attacks of singultus, and towards the last of his illness there were chills and *heats*. His pulse continued regular, and at 80 to 90 till within a few days of his death, when it arose to 120 to 150. The chills became more frequent, deglutition more difficult, respiration more distressed and painful—till they finally ceased on the 2nd of March, at 12 o'clock, M.

This is a hasty and imperfect sketch of the case; though the mere outline of it, there was such monotony in the case, such gradual decline, and such variety in the treatment, that volumes would be required to describe them all; and yet it would be little else than the history of one day, and the exhibiting of all the articles in the Materia Medica.

Everything was tried, known to the art of medicine, and likely to be beneficial, and nothing with the least success or alleviation of the symptoms. Within the last week of his life, the most obvious symptoms of pulmonary disease were developed, and we were satisfied that his end was approaching.

He had requested that his body should be examined after his death; accordingly, 24 hours after death, aided by Drs. Johnston and Causten, in the presence of Dr. B. Washington, the consulting surgeon, Dr. Hall, Dr. Frailey, and my students Messrs. Bronaugh, Lewis and King, we proceeded to make the autopsy.

The body was much emaciated.

The *Chest.* We found the left lung closely adherent to the thorax; old adhesions; the lung emphysematous; the vesicles loaded with bloody serum; the bronchial tubes filled with mucus; the lung did not collapse; numerous patches of miliary tubercles in both the lobes of the lung; several calculi encysted at the apex of the lung.

*Right Lung* did not collapse; studded with tubercles in different stages, from primary tubercles to softening. These were distributed through all the lobes. In the apex of the lung there were several calculi; we found one as large as a marble; the bronchial tubes and vesicles of the lung filled with mucus.

*Heart*, healthy; bronchial glands healthy.

*Throat.*—Pharynx more pale than usual; the epithelium of the mucous coat destroyed; small apthous elevations; the pouches on each side of the larynx filled with the food he had eaten, and much enlarged. The *œsophagus* presented the same appearance. The *larynx*—great enlargement of the vocal cords; ventricles very much enlarged, that on the right side particularly. These were filled with mucus. When removed; the mucous membrane presented small elevated patches, not larger than pins' heads. The mucous membrane of the larynx and trachea reddened. *Epiglottis*—cartilage larger and more erect than usual; the glottis or rima glottidis more patulous than I have ever seen it, indeed the whole larynx was larger than I have ever seen in any case.

The *tongue* presented nothing, with the exception of the enlargement of the cryptæ and the aphthous patches at the roots. The ventricles at its roots on each side of the frænum, between it and the anterior edge of the epiglottis cartilage, very large, and also contained portions of food, &c.

*Abdomen.*—All the viscera healthy, except the kidneys—they were larger and more congested with venous blood than usual. The intestines empty and much contracted.

*Head.*—The calvarium being removed, exhibited the cerebrum healthy; cerebellum healthy; upon removing them a large quantity of sero-sanguineous fluid ran from the spinal canal. In the fossa occupied by the left lobe of the cerebellum, was situated a foreign body, having the appearance of a large hydatid, extending from the posterior inferior part of the left lobe of the cerebellum, to the junction of the pons varolii to the crura cerebri, pressing on the left inferior side of the cerebellum, pons varolii and medulla oblongata, where the 6th, 7th, 8th and 9th pairs of nerves pass off, making a deep indentation, if not having produced absorption, into that portion of the cerebral mass. This tumor was fully as large as a hen's egg. We carefully removed it with the temporal bone, so that we could examine it properly—in doing so, we unavoidably cut into the sac, and found the contents to be a semi-transparent gelatinous substance, of the consistence of the vitreous humour of the eye, having minute red vessels passing through it.

This tumor seems to have sprung from the bone, was situated behind the dura mater, which formed on its cerebral face a thick envelope. This substance, as far as we could judge, sprang from the internal meatus, then expanded behind the dura mater, filling up the fossa immediately behind the petrous portion of the temporal bone, then passing through the posterior foramen lacerum along with the the jugular vein, par vagum nerve, passing to the edge of the foramen magnum occipitis, which seems to have formed its boundary posteriorly. Another portion of this tumor passed through the carotid foramen with the artery, then uniting with that portion which passed out of the jugular foramen, enveloping the nerve, artery and vein, and embracing the 9th pair of nerves as it passed on its way to the tongue; these, in a word, were all involved in the tumor. The tumor then extended upwards under the zygoma into the spheno-maxillary fissure surrounding the articulation of the lower jaw, then extending downwards towards the thyroid cartilage. Upon removing this tumor from the bone as far as was practicable, we found that it closely adhered to the bone internally behind the dura mater, that the bone where the tumor was situated was much thinned, readily breaking and diaphonous, that a ledge or rima of the bone was found defining the extent of the tumor, immediately posterior to the petrous portion of the temporal bone, in what is termed the mastoid portion of the bone.

Upon sawing through the petrous portion into the cavity of the ear, we found the tympanum, vestibule, and all the canals, as well as the mastoid and other cells, filled with the same substance as that which composed the tumor. The membrane lining the cavities peeled off readily; the cavity of the tympanum was filled with pus; fragments of the bones of the ear were detected; the lining membrane was entirely destroyed. The exit of this pus was prevented by a plug of hardened wax, which was situated immediately at the entrance of the cavity of the tympanum.

There conld be detected no vestige of the portio mollis except its root. The portio dura was seen wending its way to the stylo-mastoid foramen, where it was found, but soon it became involved in this tumor. We were at a loss to decide where the portion of the tumor, which protruded externally, derived its covering; it seemed to be of the same character as that which occupied the internal portion, which was evidently the dura mater. We could not make out any definite proper covering to the substance of the tumor, but I feel satisfied that it was covered with a proper coat, independent of the fibrous covering, and it had a reticulated or cellular arrangement similar to the hyaloid membrane of the vitreous humor, for the substance presented that degree of consistence, for which I could assign no other cause.

This is a most satisfactory autopsy. This early history of the case would show that the origin of all the patient's sufferings was in the ear—that all his symptoms of paralysis of the pharynx, tongue, &c., arose from the pressure of the 9th nerve. The pressure on the par vagum and sympathetic accounts for the early embarrassment of the lungs, heart and stomach. The pressure on the left side of the cerebellum, pons varolii and medulla oblongata, from which these nerves spring, causing paralysis on the right side. The difficulty of deglutition arose from the loss of nervous power and influence to the pharynx. The pain (not a severe sharp pain) about the mastoid portion of the head along down the neck, arising from the pressure of the nerves of the neck by the tumor. The lodgment of the food arising from the loss of power in the pharyngeal muscles to propel the morsel; and the loss of power to raise the larynx, also arising from the same.—*Boston Med. & Surg. Jour.*

